# Do cognitively based medical selection assessment scores predict doctors’ clinical competency? A protocol for a systematic review

**DOI:** 10.1136/bmjopen-2025-104028

**Published:** 2025-08-25

**Authors:** Taha Khan, Karen Mattick, Paul Alexander Tiffin

**Affiliations:** 1 Hull York Medical School Centre for Health and Population Sciences, York, England, UK; 2 Medical Education, University of Exeter Medical School, Exeter, UK; 3 Health Sciences, University of York, York, UK

**Keywords:** MEDICAL EDUCATION & TRAINING, Clinical Competence, Health policy, Health Workforce, Systematic Review

## Abstract

**Abstract:**

**Introduction:**

Internationally, medical schools increasingly use cognitively based selection assessments to select applicants. These tests evaluate cognitive performance and show some predictive validity for academic attainment during medical school, often incremental to that provided by secondary school grades. However, their use imposes burdens on applicants and institutions. They may also disadvantage certain under-represented groups. Therefore, to justify their adoption, these assessments should ideally predict doctors’ future clinical competency, which can be evaluated by clinical outcomes or performance in post-qualification practical clinical examinations. Hence, this systematic review aims to collate and appraise evidence linking scores from these assessments to doctors’ clinical competency.

**Methods and analysis:**

A comprehensive search strategy, co-developed with stakeholders, will search eight databases and grey literature from January 2000 onwards. Study selection, data extraction, study quality and the risk of bias assessment will be performed independently by two authors. A narrative synthesis will be used to appraise and integrate the findings from the included studies.

**Ethics and dissemination:**

Ethical approval is not required. The results will be published in a peer-reviewed journal and presented at relevant academic conferences.

**PROSPERO registration number:**

The protocol was registered prospectively on PROSPERO (CRD42024539112).

**STRENGTHS AND LIMITATIONS OF THIS STUDY**

- The protocol was prospectively registered on PROSPERO and followed the PRISMA-P (Preferred Reporting Items for Systematic Reviews and Meta-analyses Protocol) guideline.
- Patient and public involvement informed the study.
- A robust search strategy included the grey literature and was co-developed with stakeholders and peer-reviewed by an information scientist and librarians.
- Two authors will independently perform study selection, data extraction, study quality and risk of bias assessment.
- Only studies written in English will be included due to the time and costs associated with ensuring high-quality translation.

## Introduction

 Selection is important when choosing future doctors, given the power and responsibility that often accompany their role. To complicate matters, different stakeholders may prioritise different traits (the ‘criterion problem’).^1^ For example, patients often emphasise personal qualities, such as empathy and compassion. However, medical schools may prioritise students with the academic ability to pass their assessments.^2^ Moreover, internationally, the number of applicants to medical schools generally far exceeds the number of available places. Hence, admission tutors are often faced with the task of having to choose between similarly academically high-achieving candidates. Additionally, pre-university educational attainment is closely associated with socioeconomic status.^3^ Therefore, increasingly, medical schools have adopted cognitively based selection assessments.

Cognitively based medical selection assessments are aptitude tests that evaluate cognitive skills, such as problem-solving and semantic knowledge. They produce scalar metrics (scores) of performance, which are then used by selectors in a variety of ways to support and defend their decisions.^4 5^ Examples of cognitively based medical selection assessments include the University Clinical Aptitude Test (UCAT), widely used in Europe and Australasia, and the Medical College Admission Test (MCAT), used in North America.

These selection assessments were hoped to be less sensitive to sociodemographic characteristics, widening access to medicine. Indeed, early findings suggested that the robust use of cognitively based selection assessments during selection to medical school reduced the disadvantage faced by some underrepresented groups.^6^ There was also some evidence that they may be less sensitive to socioeconomic status, in comparison to secondary school grades.^7^ However, these results were not supported by subsequent longitudinal studies.^8 9^

Cognitively based selection assessments have also faced other criticisms. Serious errors have occurred in their administration.^10^ Separately, concerns have been raised regarding: their fairness; the added financial burden, time and stress imposed on applicants and their limited relevance to real-world medical practice.^11^,^14^

Thus, to justify their use, the scores from such selection assessments should demonstrate incremental predictive validity for relevant outcomes. Specifically, they should predict aspects of future performance, important to the effective study and practice of medicine, above and beyond that already provided by other selection metrics, notably secondary school grades.^15^ There is already some evidence that the scores from such selection assessments can, at least modestly, incrementally predict performance during medical school.^16^,^20^ These findings provide some reassurance of the added value of such tests.

However, individuals are admitted to medical school not just to study medicine, but to practise it. Hence, ideally, the scores from such assessments should predict doctors’ post-qualification clinical competency or at least plausible proxies.^21^,^23^ Following their review, Epstein and Hundert defined clinical competency as: ‘the habitual and judicious use of communication, knowledge, technical skills, clinical reasoning, emotions, values and reflection in daily practice for the benefit of the individual and the community being served’.^24^ For the purpose of this review, objective measures of clinical outcomes would be the preferred validity criteria for clinical competency. However, obtaining such data is extremely challenging. Moreover, it is difficult to account for the effects of potential confounding variables, such as access to resources and workload. Therefore, performance in post-qualification practical clinical examinations is likely the best readily available proxy for an individual doctor’s clinical competency. These examinations typically involve high-fidelity clinical simulations that are structured and standardised, at least to some extent, in scoring and content. Furthermore, there is strong evidence that a doctor’s performance in post-qualification clinical examinations predicts actual clinical outcomes, such as mortality rate^25 26^ and sanctions on a doctor’s licence to practise.^27 28^

**Figure 1:**
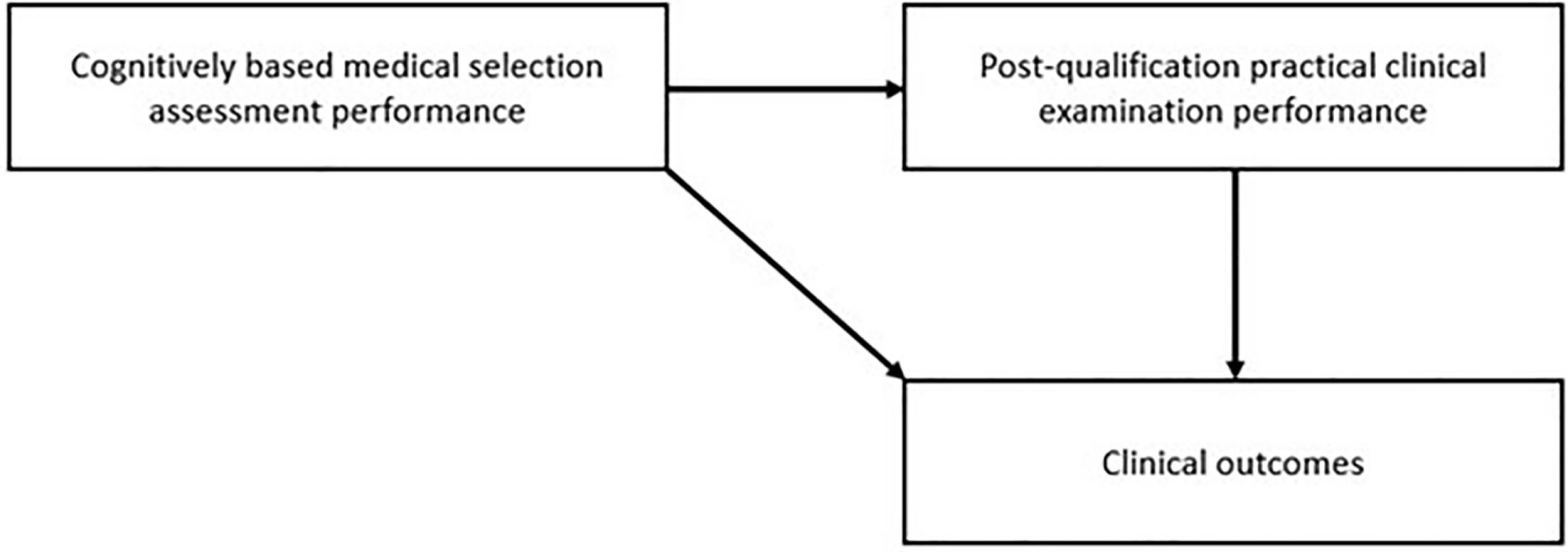
A directed acyclic graph hypothesising the causal relationships between: cognitively based medical selection assessment performance, post-qualification practical clinical examination performance and clinical outcomes.

More recent studies have explored whether performance in cognitively based medical selection assessments predicts performance in post-qualification practical clinical examinations. In the UK, Paton *et al*^23^ found that scores from the UCAT and BioMedical Admissions Test had statistically significant incremental predictive validity for the Membership of the Royal College of Physicians Practical Assessment of Clinical Examination Skills. In the USA, the MCAT has been shown to predict doctors’ performance in post-qualification medical board exams across various specialties.^29^ Additionally, performance in North American post-qualification written, knowledge-based board exams has demonstrated predictive validity for clinical outcomes. The clinical outcomes included markers of evidence-based clinical practice in primary care^30 31^ and reduced patient mortality in secondary care.^25^ These associations hold after adjusting for several potential confounders. These findings provide indirect evidence that cognitively based medical selection assessments may predict doctors’ post-qualification practical clinical examination performance, which, in turn, may predict clinical outcomes. The postulated causal relationship between these constructs is depicted in a directed acyclic graph (figure 1).

Despite these findings, important knowledge gaps remain. For example, it remains unclear how well these selection assessments predict post-qualification clinical competency and how their predictive validity varies with different tests (such as MCAT vs UCAT) and different specialties (such as surgery vs psychiatry).

Our patient and public involvement and stakeholder engagement confirmed that, since medical education is a resource-intensive and demanding endeavour,^32 33^ improving selection would be advantageous for: medical schools, applicants, the healthcare workforce and patients. Therefore, it is timely and essential to address these gaps to justify and inform their global application. Hence, this systematic review aims to identify, appraise and synthesise the relevant existing evidence on the predictive validity of cognitively based medical selection assessments for doctors’ clinical competency, including post-qualification practical clinical examination performance and clinical outcomes. By evaluating the strength and consistency of the evidence, this review will inform policy and practice in medical selection, highlight gaps in the existing literature and propose directions for future research.

## Methods and analysis

### Patient and public involvement statement

Patients, the public and stakeholders were involved in shaping the focus of this systematic review. Through engagement with key stakeholders, including medical educators, policymakers and patient representatives, we confirmed that improving medical selection is a priority given the resource-intensive and demanding nature of medical education.^32 33^ Their input reinforced the need to address gaps in evidence to justify and inform the global application of selection assessments. However, patients and the public will not be directly involved in the conduct, reporting or dissemination.

### Study design, protocol and dates

Given the focused research question, a systematic review will be used to identify and collate relevant evidence.^34^ The protocol was registered prospectively on PROSPERO (CRD42024539112).^35^ The anticipated study dates are from May 2024 to September 2025.

### Search strategy

Preliminary searches already identified some studies relevant to the research question. The MEDLINE search strategy in the online supplemental appendix was developed with the help of a health sciences librarian experienced in systematic review searching, stakeholder input (including doctors and medical educators) and an information scientist, who performed peer review using the Peer Review of Electronic Search Strategies guidelines.^36^ This ensured all pre-identified studies would be retrieved using a single search strategy.^37^ Next, the search strategy was peer-reviewed by a second independent librarian.^37 38^

The following databases will be searched: MEDLINE (OVID interface), Embase (OVID interface), American Psychological Association, PsycINFO (OVID interface), Education Resources Information Center, Scopus, Cochrane, Google Scholar and Web of Science.^39^ The grey literature will also be searched using the UCAT website, government reports, EthOS (British Library), ProQuest Dissertations & Theses Global and PROSPERO. The MEDLINE search strategy (online supplemental appendix) will be adapted to the syntax and subject headings of the other databases. Furthermore, to ensure a comprehensive search, forward searching of citing studies and backward searching of cited studies will also be performed.^40^ Finally, a bibliography of the included studies will be sent to experts in the field, such as those who have contributed the most value, for their feedback; for example, if they recommend including additional studies missed by the search strategy.

### Eligibility criteria

Studies will be deemed eligible if they evaluated the predictive validity of cognitively based medical selection assessment scores for doctors’ clinical competency, including performance in post-qualification practical clinical examinations or clinical outcomes. The full inclusion and exclusion criteria are presented in table 1.

**Table 1:** Inclusion and exclusion criteria

Characteristic	Inclusion criteria	Exclusion criteria
Population/ setting	Applicants to medical school and qualified doctors, internationally	Applicants to courses other than medicine
Predictor	Cognitively based medical selection assessment scores	Other types of selection assessments, for example, interviews
Outcomes	Performance in post-qualification practical clinical examinations or actual clinical outcomes	Post-qualification clinical examinations with only written components or self-assessed outcomes
Study design	Randomised controlled trials, prospective and retrospective cohort studies, or case-control studies	Cross-sectional, case series, case reports, qualitative, incomplete studies or non-empirical literature (eg, editorials and commentaries)
Time frame	Studies published since 2000, inclusive	Studies published before 2000
Language	Studies reported in the English language	Studies reported in other languages due to the time and costs associated with ensuring high-quality translation

The year 2000 was chosen as the cut-off for included studies as the supervisory team agreed that this was long enough to retrieve sufficient studies to analyse in the review and short enough for the results to be relevant to modern-day medical selection, reflecting the perpetually evolving landscape.

### Study selection

Retrieved studies will be uploaded and managed using Covidence. This will facilitate the review process by providing tools for classifying and labelling studies.^41^ Two authors will perform the selection of retrieved studies by independently reviewing the titles and abstracts and then full texts, based on the inclusion and exclusion criteria. Disagreements or uncertainties will be addressed by discussion among all the authors to reach a consensus.

### Data extraction

The method for data extraction will be developed a priori with consensus from all authors. The data extraction form will be evaluated using studies identified by preliminary searches. Two authors will independently perform data extraction.^42^ Disagreements or ambiguities will be addressed by discussion until a consensus is reached. The extracted information will include: study design, sample size, publication status, study population, selection assessment, primary outcome, main results, key limitations and financial support.^37^

### Risk of bias in individual studies

The Quality In Prognosis Studies (QUIPS) risk of bias tool is bespoke for studies of prognostic factors.^43^ Therefore, it was deemed suitable to assess the validity and potential bias in the results of the studies identified. The six domains of the QUIPS include: participation (representativeness of the participants from the population they draw from); attrition (the extent to which data are missing); prognostic factor measurement (the reliability, consistency and comparability of the predictors); confounding (whether these will be measurable and adjusted for); outcome measurement (reliability, consistency and comparability) and analysis and reporting (the appropriateness of the analysis and adequacy of reporting). Each domain’s risk of bias will be rated as: *high*, *moderate* or *low*. Although QUIPS Cohen’s kappa coefficient for inter-rater reliability was generally ‘substantial’,^43 44^ two authors will independently assess the risk of bias. Disagreements will be addressed by discussion until a consensus is reached.

### Synthesis

A systematic descriptive summary of the characteristics of the primary research identified will include the: population characteristics, selection assessment used, outcome(s) measured, study design, analyses, risk of bias and key limitations. A narrative synthesis will be used to appraise and integrate the findings from the included studies. This will include making judgments about the body of evidence, considering both internal and external validity and determining how the results should be interpreted.

## Ethics and dissemination

This study did not involve human participants or require access to confidential data. Therefore, ethical approval was not necessary.

Findings from this review will be published in a peer-reviewed journal, presented at relevant academic conferences and shared on social media.

## Supplementary material

10.1136/bmjopen-2025-104028 online supplemental file 1

## References

[1] Patterson F, Knight A, Dowell J (2016). How effective are selection methods in medical education? A systematic review. Med Educ.

[2] Cleland J, Blitz J, Cleutjens K (2023). Robust, defensible, and fair: The AMEE guide to selection into medical school: AMEE Guide No. 153. Med Teach.

[3] Mwandigha LM, Tiffin PA, Paton LW (2018). What is the effect of secondary (high) schooling on subsequent medical school performance? A national, UK-based, cohort study. BMJ Open.

[4] Kreiter CD, Axelson RD (2013). A perspective on medical school admission research and practice over the last 25 years. Teach Learn Med.

[5] Greatrix R, Dowell J (2020). UKCAT and medical student selection in the UK - what has changed since 2006?. BMC Med Educ.

[6] Tiffin PA, Dowell JS, McLachlan JC (2012). Widening access to UK medical education for under-represented socioeconomic groups: modelling the impact of the UKCAT in the 2009 cohort. BMJ.

[7] Tiffin PA, McLachlan JC, Webster L (2014). Comparison of the sensitivity of the UKCAT and A Levels to sociodemographic characteristics: a national study. BMC Med Educ.

[8] Mathers J, Sitch A, Parry J (2016). Longitudinal assessment of the impact of the use of the UK clinical aptitude test for medical student selection. Med Educ.

[9] Fielding S, Tiffin PA, Greatrix R (2018). Do changing medical admissions practices in the UK impact on who is admitted? An interrupted time series analysis. BMJ Open.

[10] Cassidy J (2008). UKCAT among the pigeons. BMJ.

[11] Dhar D, Perry WRG, Poole P (2012). Students’ perceptions of the Undergraduate Medicine and Health Sciences Admissions Test (UMAT). *N Z Med J*.

[12] Kelly ME, Gallagher N, Dunne FP (2014). Views of doctors of varying disciplines on HPAT-Ireland as a selection tool for medicine. Med Teach.

[13] Fortin Y, Kulasegaram KM, Kancir JN (2018). The Direct Economic and Opportunity Costs of the Medical College Admissions Test (MCAT) for Canadian Medical Students. MedEdPublish (2016).

[14] Kumar K, Roberts C, Bartle E (2018). Testing for medical school selection: What are prospective doctors’ experiences and perceptions of the GAMSAT and what are the consequences of testing?. *Adv in Health Sci Educ*.

[15] McManus IC, Ferguson E, Wakeford R (2011). Predictive validity of the Biomedical Admissions Test: an evaluation and case study. Med Teach.

[16] Dunleavy DM, Kroopnick MH, Dowd KW (2013). The predictive validity of the MCAT exam in relation to academic performance through medical school: a national cohort study of 2001-2004 matriculants. Acad Med.

[17] Puddey IB, Mercer A (2014). Predicting academic outcomes in an Australian graduate entry medical programme. BMC Med Educ.

[18] Tiffin PA, Mwandigha LM, Paton LW (2016). Predictive validity of the UKCAT for medical school undergraduate performance: a national prospective cohort study. BMC Med.

[19] Bala L, Pedder S, Sam AH (2022). Assessing the predictive validity of the UCAT-A systematic review and narrative synthesis. Med Teach.

[20] Hanson JT, Busche K, Elks ML (2022). The Validity of MCAT Scores in Predicting Students’ Performance and Progress in Medical School: Results From a Multisite Study. Acad Med.

[21] Kane MT (1992). An argument-based approach to validity. Psychol Bull.

[22] Cook DA, Brydges R, Ginsburg S (2015). A contemporary approach to validity arguments: a practical guide to Kane’s framework. Med Educ.

[23] Paton LW, McManus IC, Cheung KYF (2022). Can achievement at medical admission tests predict future performance in postgraduate clinical assessments? A UK-based national cohort study. BMJ Open.

[24] Epstein RM, Hundert EM (2002). Defining and assessing professional competence. JAMA.

[25] Norcini JJ, Lipner RS, Kimball HR (2002). Certifying examination performance and patient outcomes following acute myocardial infarction. Med Educ.

[26] Norcini JJ, Boulet JR, Opalek A (2014). The Relationship Between Licensing Examination Performance and the Outcomes of Care by International Medical School Graduates. Acad Med.

[27] Tiffin PA, Paton LW, Mwandigha LM (2017). Predicting fitness to practise events in international medical graduates who registered as UK doctors via the Professional and Linguistic Assessments Board (PLAB) system: a national cohort study. BMC Med.

[28] Wakeford R, Ludka K, Woolf K (2018). Fitness to practise sanctions in UK doctors are predicted by poor performance at MRCGP and MRCP(UK) assessments: data linkage study. BMC Med.

[29] Case SM, Swanson DB (1993). Validity of NBME Part I and Part II scores for selection of residents in orthopaedic surgery, dermatology, and preventive medicine. Acad Med.

[30] Tamblyn R, Abrahamowicz M, Brailovsky C (1998). Association between licensing examination scores and resource use and quality of care in primary care practice. JAMA.

[31] Tamblyn R, Abrahamowicz M, Dauphinee WD (2002). Association between licensure examination scores and practice in primary care. JAMA.

[32] Brazeau CMLR, Shanafelt T, Durning SJ (2014). Distress among matriculating medical students relative to the general population. Acad Med.

[33] Department of Health (2017). Expansion of undergraduate medical education: a consultation on how to maximise the benefits from the increases in medical student numbers.

[34] Grant MJ, Booth A (2009). A typology of reviews: an analysis of 14 review types and associated methodologies. *Health Info Libraries J*.

[35] Khan T, Tiffin P, Mattick K (2024). Do medical schools’ cognitively based selection assessment scores predict doctors’ post-qualification clinical competency? a protocol for a systematic review. https://www.crd.york.ac.uk/prospero/display_record.php?ID=CRD42024539112.

[36] McGowan J, Sampson M, Salzwedel DM (2016). PRESS Peer Review of Electronic Search Strategies: 2015 Guideline Statement. J Clin Epidemiol.

[37] Shamseer L, Moher D, Clarke M (2015). Preferred reporting items for systematic review and meta-analysis protocols (PRISMA-P) 2015: elaboration and explanation. BMJ.

[38] Sampson M, McGowan J (2006). Errors in search strategies were identified by type and frequency. J Clin Epidemiol.

[39] Lawrence DW (2008). What is lost when searching only one literature database for articles relevant to injury prevention and safety promotion?. Inj Prev.

[40] Greenhalgh T, Peacock R (2005). Effectiveness and efficiency of search methods in systematic reviews of complex evidence: audit of primary sources. BMJ.

[41] Babineau J (2014). Product Review: Covidence (Systematic Review Software). *J Can Health Libr Assoc*.

[42] Buscemi N, Hartling L, Vandermeer B (2006). Single data extraction generated more errors than double data extraction in systematic reviews. J Clin Epidemiol.

[43] Hayden JA, van der Windt DA, Cartwright JL (2013). Assessing bias in studies of prognostic factors. Ann Intern Med.

[44] Landis JR, Koch GG (1977). The measurement of observer agreement for categorical data. Biometrics.

