# Supplementary material for: Do cognitively based medical selection assessment scores predict doctors’ clinical competency? A protocol for a systematic review
Source: BMJ Open. 2025 Aug 25;15(8):e104028. doi: 10.1136/bmjopen-2025-104028 (PMC12382573; doi:10.1136/bmjopen-2025-104028)
Supplement: online supplemental file 1 [file bmjopen-15-8-s001.docx]

Supplementary Material: Appendix and PRISMA-P Checklist

# Appendix

| MEDLINE literature search strategy using OVID interface | |
| --- | --- |
| Concept 1: Participants | medical student* [Title/Abstract] (52594) OR  applicant* [Title/Abstract] (11441) OR  doctor* [Title/Abstract] (151866) OR  registrar* [Title/Abstract] (4881) OR  consultant* [Title/Abstract] (25742) OR  resident* [Title/Abstract] (236283) OR  attending* [Title/Abstract] (101810) OR  physician* [Title/Abstract] (464022) OR  surgeon* [Title/Abstract] (251305) OR  general practitioner* [Title/Abstract] (57394) OR  gp [Title/Abstract] (50083) OR  hospitalist* [Title/Abstract] (3043) OR  psychiatrist* [Title/Abstract] (29277) OR  obstetrician* [Title/Abstract] (17407) OR  gynaecologist* [Title/Abstract] (4192) OR  urologist* [Title/Abstract] (14325) OR  radiologist* [Title/Abstract] (64820) OR  pathologist* [Title/Abstract] (44576) OR  anaesthetist* [Title/Abstract] (7061) OR  intensivist* [Title/Abstract] (4669) OR  Students, Medical/ [MeSH Terms] (44504) OR  explode education, medical/ [MeSH Terms] (185269) OR  explode Physicians/ [MeSH Terms] (178083) |
| Concept 2:  Predictors | aptitude test* [Title/Abstract] (589) OR  admission test* [Title/Abstract] (848) OR  admission* test* [Title/Abstract] (194) OR  selection assessment* [Title/Abstract] (154) OR  UKCAT [Title/Abstract] (54) OR  UCAT [Title/Abstract] (30) OR  BMAT [Title/Abstract] (167) OR  GAMSAT [Title/Abstract] (20) OR  HPAT [Title/Abstract] (54) OR  MCAT [Title/Abstract] (901) OR  Test for Medical Studies [Title/Abstract] (8) OR  MediTest-EU [Title/Abstract] (0) OR  focus Aptitude Tests/ [MeSH Terms] (935) |
| Boolean operators | The concepts were combined using the Boolean operators:  1 AND 2 |

# PRISMA-P Checklist

| Administrative information | |
| --- | --- |
| Title | Do cognitively based medical selection assessment scores predict doctors’ clinical competency? A protocol for a systematic review |
| Registration | The protocol was registered prospectively on PROSPERO (CRD42024539112) |
| Authors | Corresponding author: Dr Taha Khan^1^ taha.khan6@nhs.net  Professor Karen Mattick^1^ k.l.mattick@exeter.ac.uk  Guarantor: Professor Paul Tiffin^2^ paul.tiffin@york.ac.uk  ^1^University of Exeter Medical School, St Luke's Campus, Heavitree Road, Exeter EX1 2LU.  ^2^Hull York Medical School, Department of Health Sciences, Siwards Way, York YO10 5DD.  TK wrote the protocol. PT and KM recommended edits. All authors read and approved this final version. |
| Amendments | Not applicable |
| Support | This research was supported by the University Clinical Aptitude Test (UCAT). The lead author (TK) undertook the work as part of a National Institute of Health Research (NIHR) funded Academic Clinical Fellowship. However, neither of these organisations is the sponsor as they were not involved in any aspect of the project, such as the protocol, analysis, interpretation or publication. Hence, the views expressed are those of the authors and not necessarily those of the UCAT, NIHR or the Department of Health and Social Care. |
| Introduction | |
| Rationale | Internationally, medical schools increasingly use cognitively based selection assessments to select applicants. These tests evaluate cognitive performance and show some predictive validity for academic attainment during medical school, often incremental to that provided by secondary school grades. However, their use imposes burdens on applicants and institutions. They may also disadvantage certain underrepresented groups. Therefore, to justify their adoption, these assessments should ideally predict doctors’ future clinical competency, which can be evaluated by clinical outcomes, or performance in post-qualification practical clinical examinations. |
| Objective | Hence, this systematic review aims to collate and appraise evidence linking scores from these assessments to doctors’ clinical competency, including clinical outcomes, or performance in post-qualification practical clinical examinations. |
| Methods | |
| Eligibility criteria | Studies will be deemed eligible if they evaluated the predictive validity of cognitively based medical selection assessment scores for doctors’ clinical competency, including performance in post-qualification practical clinical examinations or clinical outcomes. The full inclusion and exclusion criteria are presented in Table 1. |
| Information sources | The following databases will be searched: MEDLINE (OVID interface), Embase (OVID interface), American Psychological Association PsycINFO (OVID interface), Education Resources Information Center, Scopus, Cochrane, Google Scholar, and Web of Science. The grey literature will also be searched using the UCAT website, government reports, EthOS (British Library), ProQuest Dissertations & Theses Global, and PROSPERO. |
| Search strategy | See Appendix |
| Study records | Retrieved studies will be uploaded and managed using Covidence. This will facilitate the review process by providing tools for classifying and labelling studies. Two authors will perform the selection of retrieved studies by independently reviewing the titles and abstracts, and then full texts, based on the inclusion and exclusion criteria. Disagreements or uncertainties will be addressed by discussion amongst all the authors to reach a consensus.  The method for data extraction will be developed a priori with consensus from all authors. The data extraction form will be evaluated using studies identified by preliminary searches. Two authors will independently perform data extraction. Disagreements or ambiguities will be addressed by discussion until a consensus is reached. |
| Data items | The extracted information will include: study design, sample size, population, publication status, selection assessment, outcome, analysis, main results, key limitations and financial support. |
| Outcomes and prioritisation | The outcomes for doctors’ clinical competency will include performance in post-qualification practical clinical examinations or clinical outcomes. Clinical outcomes will be prioritised as these are preferred measures of competency, whereas performance in post-qualification practical clinical examinations serve as the best proxy. Nevertheless, if a study reports both outcomes, both will be discussed in the review. |
| Risk of bias in individual studies | The Quality In Prognosis Studies (QUIPS) risk of bias tool is bespoke for studies of prognostic factors. Therefore, it was deemed suitable to assess the validity and potential bias in the studies identified. |
| Data synthesis | A narrative synthesis will bring together the data in a way that answers the research question. Judgments will be made about the body of evidence, including the internal and external validity, and how these impact interpretation.  Results will be summarised using appropriate summary statistics. If a meta-analysis is appropriate, this will include a robust quantitative synthesis to determine the direction, size and consistency of the predictive power of these selection assessments on post-qualification practical clinical examination performance and clinical outcomes. If data is missing, the original study authors will be emailed to request the data (as this method of communication is reported to have the greatest response rate in the shortest time with the fewest attempts needed). If unsuccessful, imputation will be implemented, followed by sensitivity analyses to evaluate the potential impact on the study findings. |
| Meta-bias | Meta-bias is a systematic error in the design of a meta-analysis, including the identification and analysis of individual studies, rather than bias within the studies themselves. Meta-bias comes in 3 main forms: selection bias, information bias, and analysis bias.  Selection bias includes reporting bias (such as publication bias, selective outcome bias, and ascertainment bias) and inclusion bias. To assess publication bias (that research with statistically significant results is published more often, more quickly, in journals with higher impact factors, and in the English language, than research with statistically insignificant results) the grey literature will be searched. In addition, if appropriate, a funnel plot will be created, and Egger’s test will be calculated. To assess selective outcome reporting, studies will be compared with their protocols, if available.  Inclusion bias is a systematic error in the inclusion of studies for a systematic review that favours one outcome over another. It can be considered a type of selection bias or information bias. An example is if search strategies are tailored to specifically include or exclude certain studies since their outcomes are already known. To mitigate inclusion bias, the search strategy has been peer-reviewed. In addition, selection will be performed by two reviewers, independently.  To mitigate information bias (the systematic distortion of selecting, extracting or appraising information to match a certain narrative), none of the authors had a conflict of interest, and two reviewers will complete selection and data extraction independently. In addition, missing data will be addressed by contacting the study author. Blinding of certain study details (such as authors, institution, or journal) will not be performed as it has not demonstrated sufficient value in other studies, and is too labour-intensive.  To enable readers to assess analysis bias, the intended analyses have been reported transparently and justified in the protocol. |
| Confidence in cumulative evidence | A formal GRADE assessment is not planned, due to the observational nature of the included studies. Nevertheless, the strength of evidence will be assessed by considering the QUIPS risk of bias domains alongside the overall consistency, directness, precision, and potential meta-bias of the studies. Judgements will be qualitatively summarised, focusing on both internal and external validity, and how these factors affect the interpretation of the findings. |
