# Supplementary material for: Do cognitively based medical selection assessment scores predict doctors’ clinical competency? A protocol for a systematic review
Source: BMJ Open. 2025 Aug 25;15(8):e104028. doi: 10.1136/bmjopen-2025-104028 (PMC12382573; doi:10.1136/bmjopen-2025-104028)
Supplement: Supplementary file 2 [file bmjopen-2025-104028.reviewer_comments.pdf]

PEER REVIEW HISTORY

BMJ Open publishes all reviews undertaken for accepted manuscripts. Reviewers are asked to complete a checklist review form and are provided with free text boxes to elaborate on their assessment. These free text comments are reproduced below.

ARTICLE DETAILS

Title (Provisional)

Do cognitively based medical selection assessment scores predict doctors' clinical competency? A protocol for a systematic review

Authors

Khan, Taha; Mattick, Karen; Tiffin, Paul Alexander

VERSION 1 - REVIEW

**Reviewer**	**1**
**Name**	**Langhan, Melissa L.**
**Affiliation**	**Yale School of Medicine**
**Date**	**22-May-2025**
**COI**	**None**

The authors state that they are performing this review to "justify the adoption" of standardized tests used for medical school admission. As the authors note, the tests have been widely adopted and implemented for decades. Thus the driving premise for the study is flawed. The authors also seem to minimize the criticisms of standardized tests. Many institutions are moving away from scored tests, for example change to pass/fail grading, and schools and programs are shifting to more holistic assessments of applicants given the concerns with these tests.

**Reviewer**	**2**
**Name**	**Ernawati, Desak**
**Affiliation**	**Udayana University Faculty of Medicine**
**Date**	**25-May-2025**
**COI**	**None**

Dear Editor,

Thank you for the opportunity to review this manuscript. The article will provide an important finding to the medical practice community. However, I would expect the references used to be more updated.

Reviewer 3
Name Fleming, Simon
Affiliation Queen Mary University of London, Institute of Health Sciences Education
Date 07-Jun-2025
COI None

I read this study protocol with great interest

In particular I welcome sections such as:

Despite these findings, important knowledge gaps remain. For example, it remains unclear how well these selection assessments predict post-qualification clinical competency, and how their predictive validity varies with different tests (such as MCAT versus UCAT) and different specialties (such as surgery versus psychiatry).

The fear otherwise is that this systematic review may be the tail wagging the dog. Showing that people who are good at passing specific types of exams go on to also be good at passing other exams is neither new, nor helpful. However, this protocol recognises this and goes on to explore the more nuanced facets of selection.

It is always difficult to identify proxys for future clinical competence and I wonder if the counter is true - whether low marks or initial unsuccessful attempts in these selection assessments lead to future dyscompetence

The quote that spring to mind, in the context of these section assessments are

"…assessment acts as a 'gate' – to pass an assessment may provide entry to future employment, status and other opportunity. In short: assessment is power. It legitimises knowledge and has the capacity to include, exclude and 'discipline' (to borrow Foucault's term) students and academics alike …"

Bearman, Dawson & Tai (2020)

Assessment that treats all students the same is by definition inequitable, because it ignores differences in students' past and present circumstances

I await the SR with baited breath

Reviewer 4
Name Razavinia, Fatemeh

Affiliation **Ahvaz Jondishapour University of Medical Sciences, Midwifery Department, Reproductive Health Promotion Research Center, Ahvaz Jundishapur University of Medical Sciences, Ahvaz, Iran**

Date **09-Jul-2025**

COI **None**

I appreciate the opportunity to review the manuscript titled "Do cognitively based medical selection assessment scores predict doctors' clinical competency? A protocol for a systematic review"

Abstract

1- How will the dispute be resolved in the event of a dispute between two reviewers?

2- Why the time limit and from January 2000?

Introduction

3- Explain what is meant by clinical competence and what is expected and understood after graduation.

Material and Methods

4- Page 7 lines 15 should the verb be in the future tense.

The future tense should be used in all parts because this is a study protocol and is before the study has been done. Has the study or part of it been done, please explain?

5- Only studies in English will be used, so what about studies in other languages? Write in the study restriction.

6- Why the time limit and from January 2000?

Discussion

7- A discussion should be added and the strengths and weaknesses of the study should be written in that section.

Meta-Analysis Feasibility

8- Limitations and obstacles of the study should be written and how they could affect the strength of the conclusions should be written.

Conflict of Interest and Funding Clarity

9- The study stated that UCAT and NIHR supported them but were not involved in the protocol or analyses. This should be explained clearly and precisely.

VERSION 1 - AUTHOR RESPONSE

**Feedback**	**Authors' Responses**
The authors state that they are performing this review to "justify the adoption" of standardized tests used for medical school admission. As the authors note, the tests have been widely adopted and implemented for decades. Thus the driving premise for the study is flawed.	We believe that, although the practice is already well-adopted, our review is important and necessary to discuss the empirical evidence to justify or refute the use of these tests. Indeed, it is precisely *because* these tests have been widely adopted that they require robust justification. Hence, understanding the long-term predictive validity of these tests remains crucial. Our review is also timely because empirical evidence was lacking at the time of these tests' introduction, but has developed since then.<br><br>Furthermore, we note that other peer reviewers wrote: "The article will provide an important finding to the medical practice community" and "I await the SR with baited breath". The external funding provided by a key stakeholder also demonstrates the importance of the research question. Therefore, we disagree with the reviewer who felt that the premise for the study is flawed.
The authors also seem to minimize the criticisms of standardized tests.	The criticisms of these standardised tests will not be minimised in the research. Instead, the literature will be carefully curated for evidence, to support or refute any criticisms, to inform the reader and emphasise the importance of empirical justification for their widespread adoption.<br><br>In terms of the protocol as written, we already highlight that serious errors have occurred during their administration and the concerns that have been raised regarding: their fairness; the added financial burden, time and stress imposed on applicants; and, their limited relevance to real-world medical practice. These risks and burden should be offset by evidence of substantial benefit if the use of such admissions testing is to be justified.
Many institutions are moving away from scored tests, for example change to pass/fail grading, and schools and programs are shifting to more holistic assessments of applicants given the concerns with these tests.	The claim that institutions are moving away from scored tests does not reflect the current UK landscape for medical school admissions, nor many/most international settings. Based on extensive stakeholder engagement with UK medical school admissions leads, discussions at ASME ASM 2025 (an international conference in medical education where one of the three central themes was 'assessment'), and discussions with the largest provider of cognitively based medical selection assessments (UCAT), there is no indication of a shift away from scored formats, or a decreased appetite for their use by selectors. Indeed, the UCAT has, in recent years, been adopted in some Australasian institutions, as well as additional UK-based medical schools.<br><br>This US-based peer reviewer may be referring to the recent change to USMLE Step 1 from scored to pass/fail. However, this is a completely different test (a postgraduate licensure exam assessing detailed medical

	knowledge), administered to a different population (medical students already enrolled in training), for a different purpose (regulatory progression rather than admissions). Hence, it is not relevant to the nature by which cognitively based selection assessments are scored.
How will the dispute be resolved in the event of a dispute between two reviewers?	In the existing protocol, we write: "Disagreements or uncertainties will be addressed by discussion amongst all the authors to reach a consensus".
Why the time limit and from January 2000?	The following explanation has now been added under Table 1:<br>"The year 2000 was chosen as the cut-off for included studies as the supervisory team agreed that this was long enough to retrieve enough studies to analyse in the review and short enough for the results to be relevant to modern-day medical selection, reflecting the perpetually evolving assessment landscape."
Explain what is meant by clinical competence and what is expected and understood after graduation.	After careful consideration, we have amended an introductory paragraph to provide an evidence-based definition of clinical competency:<br>"However, individuals are admitted to medical school not just to study medicine, but to practice it. Hence, ideally, the scores from such assessments should predict doctors' post-qualification clinical competency, or at least plausible proxies (21–23). Following their review, Epstein & Hundert defined clinical competency as: "the habitual and judicious use of communication, knowledge, technical skills, clinical reasoning, emotions, values, and reflection in daily practice for the benefit of the individual and the community being served" (24). For the purpose of this review, objective measures of clinical outcomes would be the preferred validity criteria for clinical competency."<br><br>A broader discussion of "what is expected and understood after graduation" falls outside the scope of this protocol.
Page 7 lines 15 should the verb be in the future tense.	We believe this peer reviewer has written this comment in error since page 7 only contains the MEDLINE search strategy. Hence, there isn't a verb on line 15 that needs changing. We have also re-read the manuscript and did not identify any other verbs that needed their tense changed.
Only studies in English will be used, so what about studies in other languages? Write in the study restriction.	As explained in Table 1, studies reported in languages other than English will be excluded. This was already acknowledged as a limitation in the section: "Strengths and limitations of this study".
A discussion should be added and the strengths and weaknesses of the study should be written in that section.	The strengths and limitations of this study have been explained in the section titled "Strengths and limitations of this study", in keeping with the journal requirements.<br>We have explained that: "A narrative synthesis will be used to appraise and integrate the findings from the included studies. This will include making judgments about the body of evidence, considering both internal and external validity, and determining how the results should be interpreted." This will form the discussion of the intended review.
Limitations and obstacles of the	Please see the explanation above.

study should be written and how they could affect the strength of the conclusions should be written.	
The study stated that UCAT and NIHR supported them but were not involved in the protocol or analyses. This should be explained clearly and precisely.	We have now provided further clarification that: "This research was *funded* by the University Clinical Aptitude Test (UCAT). The lead author (TK) undertook the work as part of a National Institute of Health Research (NIHR) funded Academic Clinical Fellowship. However, neither of these organisations is the sponsor as they will not be involved in any aspect of the project, such as the protocol, analysis, interpretation or publication. Hence, the views expressed are those of the authors and not necessarily those of the UCAT, NIHR or the Department of Health and Social Care".
